# Empagliflozin ameliorates diabetic cardiomyopathy via regulated branched-chain amino acid metabolism and mTOR/p-ULK1 signaling pathway-mediated autophagy

**DOI:** 10.1186/s13098-023-01061-6

**Published:** 2023-05-06

**Authors:** Lin Zhang, Heming Zhang, Xiuzhu Xie, Ruping Tie, Xiaolin Shang, Qianqian Zhao, Junjie Xu, Liyuan Jin, Jinying Zhang, Ping Ye

**Affiliations:** 1 Medical School of Chinese PLA, Department of Geriatric Cardiology, The Second Medical Centre, Chinese PLA General Hospital, Beijing, China; 2 Department of Anesthesiology, The 963 Hospital of the PLA Joint Logistics Support Force, Jiamusi, China; 3 Department of Pharmacy, Medical Support Center of Chinese PLA General Hospital, Beijing, China; 4 Medical School of Chinese PLA, Department of Cardiology, The Sixth Medical Centre, Chinese PLA General Hospital, Beijing, China; 5 Health Service Department of the Guard Bureau of the General Office of the Central Committee of the Communist Party of China, Beijing, China; 6 Department of Geriatric Cardiology, The Second Medical Centre, Chinese PLA General Hospital, Beijing, China; 7 Department of Basic Medicine, Medical School of Chinese PLA, Chinese PLA General Hospital, Beijing, China; 8 Department of Anesthesiology, The Second Affiliated Hospital of Air Force Medical University, Xi’an, China

**Keywords:** Empagliflozin, Diabetic cardiomyopathy, Branched-chain amino acid, Sodium–glucose co-transporter 2 inhibitor, Autophagy

## Abstract

**Background:**

Empagliflozin, a sodium–glucose co-transporter 2 inhibitor (SGLT2i), has been reported to significantly reduce the risk of heart failure in multiple clinical studies. However, the underlying mechanisms remain elusive. This study aimed to investigate the effect of empagliflozin on branched-chain amino acid (BCAA) metabolism in diabetic cardiomyopathy.

**Methods:**

Thirty male 8-week KK Cg-Ay/J mice were used to study diabetic cardiomyopathy; here, 15 were used as the model group, and the remaining 15 were administered empagliflozin (3.75 mg/kg/day) by gavage daily for 16 weeks. The control group consisted of fifteen male 8-week C57BL/6J mice, whose blood glucose and body weight were measured simultaneously with the diabetic mice until 16 weeks without additional intervention. Echocardiography and histopathology were performed to evaluate cardiac structure and function. Proteomic sequencing and biogenic analysis were performed on mouse hearts. Parallel Reaction Monitoring and western blotting were performed to validate the expression levels of differentially expressed proteins.

**Results:**

The results showed that empagliflozin improved ventricular dilatation and ejection fraction reduction in diabetic hearts, as well as the elevation of myocardial injury biomarkers hs-cTnT and NT-proBNP. At the same time, empagliflozin alleviates myocardial inflammatory infiltration, calcification foci deposition, and fibrosis caused by diabetes. The results of the proteomics assay showed that empagliflozin could improve the metabolism of various substances, especially promoting the BCAA metabolism of diabetic hearts by up-regulating PP2Cm. Furthermore, empagliflozin could affect the mTOR/p-ULK1 signaling pathway by reducing the concentration of BCAA in diabetic hearts. When mTOR/p-ULK1 protein was inhibited, ULK1, the autophagy initiation molecule, increased. Moreover, autophagy substrate p62 and autophagy marker LC3B were significantly reduced, indicating that the autophagy activity of diabetes inhibition was reactivated.

**Conclusions:**

Empagliflozin may attenuate diabetic cardiomyopathy-related myocardial injury by promoting the catabolism of BCAA and inhibiting mTOR/p-ULK1 to enhance autophagy. These findings suggest that empagliflozin could be a potential candidate drug against BCAA increase and could be used for other cardiovascular diseases with a metabolic disorder of BCAA.

## Introduction

During the past decade, the global prevalence of diabetes (mainly type 2 diabetes) has increased by 30% and is likely to increase further [1]. Approximately 451 million adults worldwide have diabetes, which could reach 693 million by 2045 [2]. Diabetic cardiomyopathy (DM) is an increasingly recognized entity, defined as a diabetic cardiac complication with ventricular dysfunction in the absence of coronary atherosclerosis and hypertension [3]. Heart failure is a common complication of diabetes, with an increased prevalence of up to 22% in patients with diabetes [4]. Therefore, the burden of diabetes-related heart failure in the healthcare system will continue to rise. Although numerous experimental and preclinical studies of DM have been conducted, no consensus has been reached regarding the most effective therapeutic approach for this disease. Empagliflozin, a selective SGLT2i, can enhance renal glucose excretion to regulate blood glucose in an insulin-independent manner [5]. Evidence suggests that empagliflozin could significantly reduce the risk of cardiovascular-related death by 38% in patients with type 2 diabetes mellitus (T2DM), with or without cardiovascular disease, and decrease the probability of myocardial infarction or stroke [6]. Empagliflozin significantly ameliorated the prognosis of heart failure independent of glucose control [7]. These findings suggest the promising potential for empagliflozin in the treatment of DM. However, the cardioprotective mechanism remains unclear.

Branched-chain amino acids (BCAAs, including leucine, isoleucine, and valine) are essential amino acids that play important roles in protein synthesis and glucose metabolism. BCAA levels are elevated in individuals with diabetes and could serve as prognostic markers for diabetes [8, 9]. Evidence suggests that the accumulation of intra-myocardial levels of BCAA catabolic mediators is a conserved metabolic signature in failing hearts [10]. Empagliflozin treatment augmented BCAA catabolism and transportation in diabetic mice [11].

The mammalian target of rapamycin (mTOR), an atypical serine/threonine kinase, is a crucial regulator of diabetic progression [12]. Studies have shown that activating the mTOR/p-ULK1 signaling pathway could alleviate diabetes-induced cardiac hypertrophy and fibrosis [8, 13]. In addition, evidence suggests that the obstacle to BCAA metabolism is associated with insulin resistance, which could regulate the mTOR/p-ULK1 signaling pathway [14].

Therefore, we aimed to determine the potential efficacy of empagliflozin in regulating BCAA metabolism and mTOR signaling to prevent diabetes-induced myocardial dysfunction.

## Methods

### Animal procedure and drug treatment

Thirty 8-week-old KK Cg-Ay/J (KK-Ay) male mice (genetic type 2 diabetes model) and fifteen 8-week-old C57BL/6J male mice were purchased from Beijing HFK Bioscience Co. Ltd. (Beijing, China). KK-Ay mice were fed a high-fat diet (HFD) with a 40% fat energy supply ratio. Blood glucose levels were measured once every three days. After 2 weeks of HFD, the following experiments were performed when the blood glucose concentration of mice at 10 weeks was stable above 13.9 mM (250 mg/dL). Subsequently, diabetic mice were randomized into two groups, the diabetic cardiomyopathy disease (DM) group, KK-Ay mice with no treatment, and the diabetic cardiomyopathy treated with Empagliflozin (DM + EMPA) group, KK-Ay mice treated with empagliflozin (Boehringer Ingelheim, Germany) 3.75 mg/kg/day for 16 weeks by gavage. The control (CTRL) group consisted of fifteen 8-week C57BL/6J mice, whose blood glucose and body weight were measured simultaneously with the diabetic mice and were fed maintenance feed until 16 weeks without additional intervention. The Ethics Committee of PLA General Hospital reviewed and approved the experiments.

### Echocardiographic evaluation

Echocardiographic measurement (Esaote Sigma Series, Italy) was performed at the end of the study period. Mice anesthetized with isoflurane were fixed at a 40 °C thermostat. A phased array fan scanning probe was used to probe the left ventricular major axis, the bottom of the minor heart axis, and the apical four-chamber view of the heart. We checked the indicators, including interventricular septal thickness in diastole (IVSd), interventricular septal thickness in systole (IVSs), Left ventricular internal diameter at end-diastole (LVIDd), Left ventricular internal diameter at end-systole (LVIDs), left ventricular posterior wall thickness at end-diastole (LVPWd), left ventricular end-systolic posterior wall thickness (LVPWs), left ventricular end-diastolic volume (LVEDV), left ventricular end-systolic volume (LVESV), left ventricle ejection fraction (EF), left ventricle fractional shortening (FS), the ratio of diastolic mitral inflow between early E to late A (E/A).

### Detection of biochemical markers in serum

The blood specimens were collected into centrifugal tubes via the orbital artery and vein from each animal, placed at room temperature for half an hour, and centrifuged at 3000 RPM for 20 min. 150 μL of serum was collected and placed in an automatic biochemical analyzer (Rayto Life, China) to detect the blood lipids of mice, triglyceride (TG), total cholesterol (TC), low-density lipoprotein (LDL), and high-density lipoprotein (HDL). Another 100 μL serum was taken to detect NT-proBNP and hs-cTnT by enzyme-linked immunosorbent assay (MEIMIAN, China). A microplate reader (ThermoFisher, USA) analyzed and quantified the plates at 450 nm and reported the results as pg/mL.

### Histomorphology

After the animal bled to death, their hearts were removed and rinsed with a PBS buffer at the end of the study. The 10 hearts in each group were frozen in liquid nitrogen and stored at − 80 °C. And 5 more hearts in each group were fixed in 4% paraformaldehyde for 48 h and embedded in paraffin cut into 3 μm thick sections. Hematoxylin and eosin (H&E) staining was used to evaluate the morphological and structural alterations in the cardiac tissue. Masson's trichrome staining was used to assess the extent of fibrosis in them. The fibrotic and cardiomyocyte cross-sectional areas (three mice per group) were measured using ImagePro PLUS software (Media Cybernetics, USA). Collagen content was presented as the collagen volume fraction (collagen area/field area × 100%).

### Proteomics

#### Protein extraction and digestion

Frozen cardiac tissue samples were transferred to a tissue grinding tube and lysed with lysis buffer, followed by sonication on ice using a homogenizer (Servicebio). Finally, the supernatant was collected after centrifugation, and the protein concentration was determined using a BCA Protein Quantitative Kit (Beyotime). The quantified protein was successively treated with dithiothreitol, iodoacetamide, and trypsin buffers.

#### Liquid chromatography–tandem mass spectrometry (LC–MS) analysis

Samples from the three groups were separated by an Easy nLC system (Thermo Fisher Scientific) with a nanoliter flow rate. LC–MS/MS analysis was performed using an Orbitrap Exploris 480 mass spectrometer (Thermo Fisher Scientific) coupled to an Easy nLC (Thermo Fisher Scientific). The peptide of each sample was desalted on C18 Cartridges, concentrated by vacuum centrifugation, and re-dissolved in 0.1% formic acid. Then, 2 μg peptide was loaded onto a C18 reversed-phase analytical column (Thermo Fisher Scientific, Acclaim PepMap RSLC 50 µm × 15 cm, nano viper, P/N164943) in buffer A (0.1% formic acid) and separated using a linear gradient of buffer B (80% acetonitrile and 0.1% formic acid) at a flow rate of 300 nL/min. The linear gradient was as follows: 4–6% B, 2 min; 6–28% B, 73 min; 28–38% B, 5 min; 38–100% B, 5 min; 100% B, 5 min.

The MS data indicated the most abundant precursor ions from the survey scan (350–1200 m/z) for HCD fragmentation. MS1 scans were acquired at a resolution of 120,000 at m/z 200, with automatic gain control (AGC) target of 300% and a maximum IT of 50 ms. Mass–charge ratios for peptides and peptide fragments were collected as follows: the data-dependent mode was set with a cycle time of 1.5 s; then, the MS2 Activation Type was set to HCD, with an isolation window of 1.6 m/z and AGC target of 75%. MS2 scans were acquired at a resolution of 15,000 at m/z 200, AGC target of 75%, maximum IT of 35 ms, and isolation width of 1.6 m/z. Microscan was set to 1. Dynamic exclusion time for selected ions was set to 30 s. The normalized collision energy was 33%.

#### Data processing, protein identification, and deposition into a public database

The acquired LC–MS/MS raw data were analyzed using SpectroMine software version 2.6.210114.47784 against the Uniprot_Musculus_17082_20210928_swissprot database. The initial search was performed using a precursor mass window of 6 ppm. The search followed the enzymatic cleavage rule of Trypsin/P and allowed two maximal missed cleavage sites and a mass tolerance of 20 ppm for fragment ions. Carbamidomethylation of cysteines was defined as a fixed modification, whereas protein N-terminal acetylation and methionine oxidation were defined as variable modifications for database searching. The global false discovery rate cutoff for peptide and protein identification was set to 0.01. Protein abundance was calculated based on the normalized spectral protein intensity. Proteins with fold change > 1.2 or < 0.83 and *P* (Student's *t*-test) < 0.05 were considered to be differentially expressed proteins. Hierarchical clustering, Gene Ontology (GO) annotation, and the Kyoto Encyclopedia of Genes and Genomes (KEGG) were utilized to analyze and classify the proteomic data.

#### Assessment of BCAA metabolism in heart tissue

The BCAA concentration was determined with a BCAA Colorimetric Assay Kit (BioVision) according to a previously described protocol. The absorbance of each sample was measured at 450 nm using a microplate reader and calculated based on a standard curve. Parallel Reaction Monitoring (PRM) Target Proteomics was used to detect BCAA metabolism-related enzymes of the three groups. Branched-chain ketoacid dehydrogenase (BCKDH), which is composed of four subunits and its regulatory proteins, is not less than 3 unique peptides (unmodified, without missing cleavage) that can be used for PRM quantitation. Mass spectrometry parameters were set as follows: (1) full-MS: scan range (m/z) = 350–1800, resolution = 60,000, AGC target = 3e6, and maximum injection time = 50 ms; and (2) PRM: resolution = 30,000, AGC target = 2e5, maximum injection time = 50 ms, loop count = 10, isolation window = 2.0 m/z, and NCE = 27 eV. Myocardial and serum samples were collected and immediately restored at − 80 °C.

#### Analysis of the mTOR and autophagy pathway by western blotting

The total protein was extracted from mouse ventricles. Aliquots containing 100 μg of protein were separated by SDS-PAGE, transferred onto a membrane, and then blocked with Quick Block blocking solution for 30 min at room temperature. Afterward, the membrane was incubated with primary antibodies overnight and appropriate secondary antibodies for 2 h. Finally, the gel image processing system analyzed the optical density of the target strip. Antibodies against p-mTOR and p-ULK1 were purchased from Abcam (Cambridge, MA, USA), and mTOR, ULK1, P62, and LC3B were purchased from ZENBIO. Anti-mouse β-actin was used as a control.

### Statistical analysis

Experimental data were presented as means ± standard error of the mean (SEM) of at least three biological replicates or independent experiments and were compared by Student's *t*-test or two-way analysis of variance (ANOVA) (when three groups were compared) by GraphPad Prism 8 software; *P* values < 0.05 were considered statistically significant.

## Results

### Effects of empagliflozin on physical and biochemical parameters of diabetic mice

**Fig. 1:**
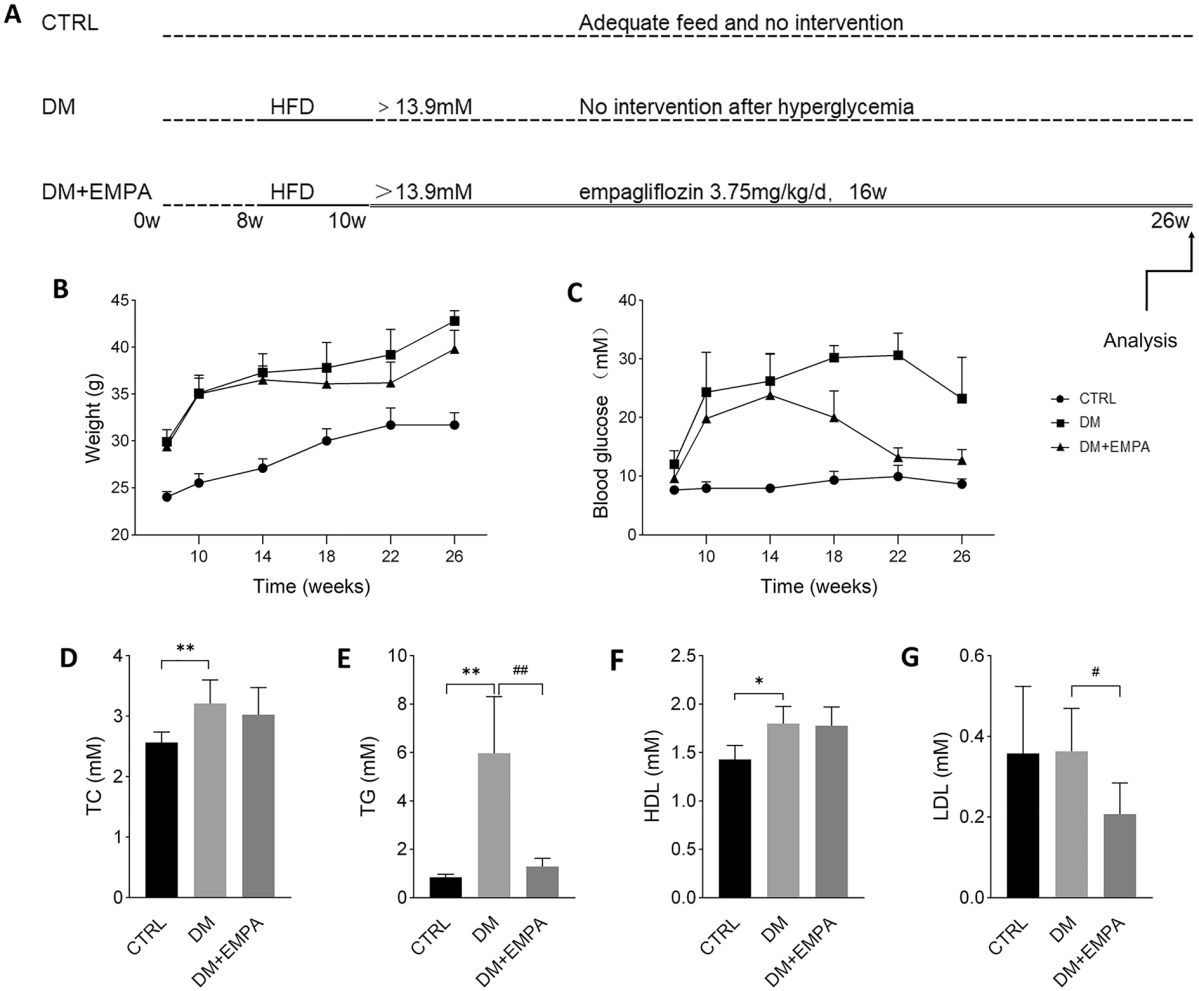
Weight and blood glucose after 16 weeks of treatment in mice. **A** Experimental flow chart. **B** Trends of body weight in mice. **C** Trends of blood glucose in mice. **D** TG: triglycerides. **E** TC: total cholesterol. **F** HDL: high-density lipoprotein cholesterol. **G** LDL: low-density lipoprotein cholesterol. Data were expressed as the mean ± SEM. *P < 0.05 versus CTRL; **P < 0.01 versus CTRL; ^#^*P* < 0.05 versus DM; ^##^*P* < 0.01 versus DM

Although KK-Ay mice were spontaneous diabetic mice, a short-term high-fat diet was still required to induce blood glucose increase. After 2 weeks of HFD induction, the random blood glucose of KK-Ay mice exceeded 13.9 mM. Sixteen weeks after intragastric administration of empagliflozin, the general conditions of the mice were evaluated, revealing that the mice in the DM group were inactive and irritable, with sparse hair and occasional arching back. Some mice were accompanied by ascites during sampling. In contrast, the DM + EMPA group showed flexible escape, smooth hair, and no ascites. At the end of the study, there were significant differences in blood glucose (CTRL vs. DM vs. DM + EMPA: 9.08 ± 0.55 vs. 30.60 ± 2.40 vs. 17.25 ± 1.39 mM, *P* < 0.01) and body weight (CTRL vs. DM vs. DM + EMPA: 31.63 ± 1.51 vs. 39.97 ± 1.30 vs. 40.45 ± 0.64, *P* < 0.01) among the three groups. The mice's body weight, blood glucose, and lipid levels are shown in Fig. 1. In our study, mice administered empagliflozin maintained relatively normal blood glucose, TG, and LDL levels compared with those in the DM group.

### Empagliflozin alleviated diabetes-induced cardiac dysfunction

**Fig. 2:**
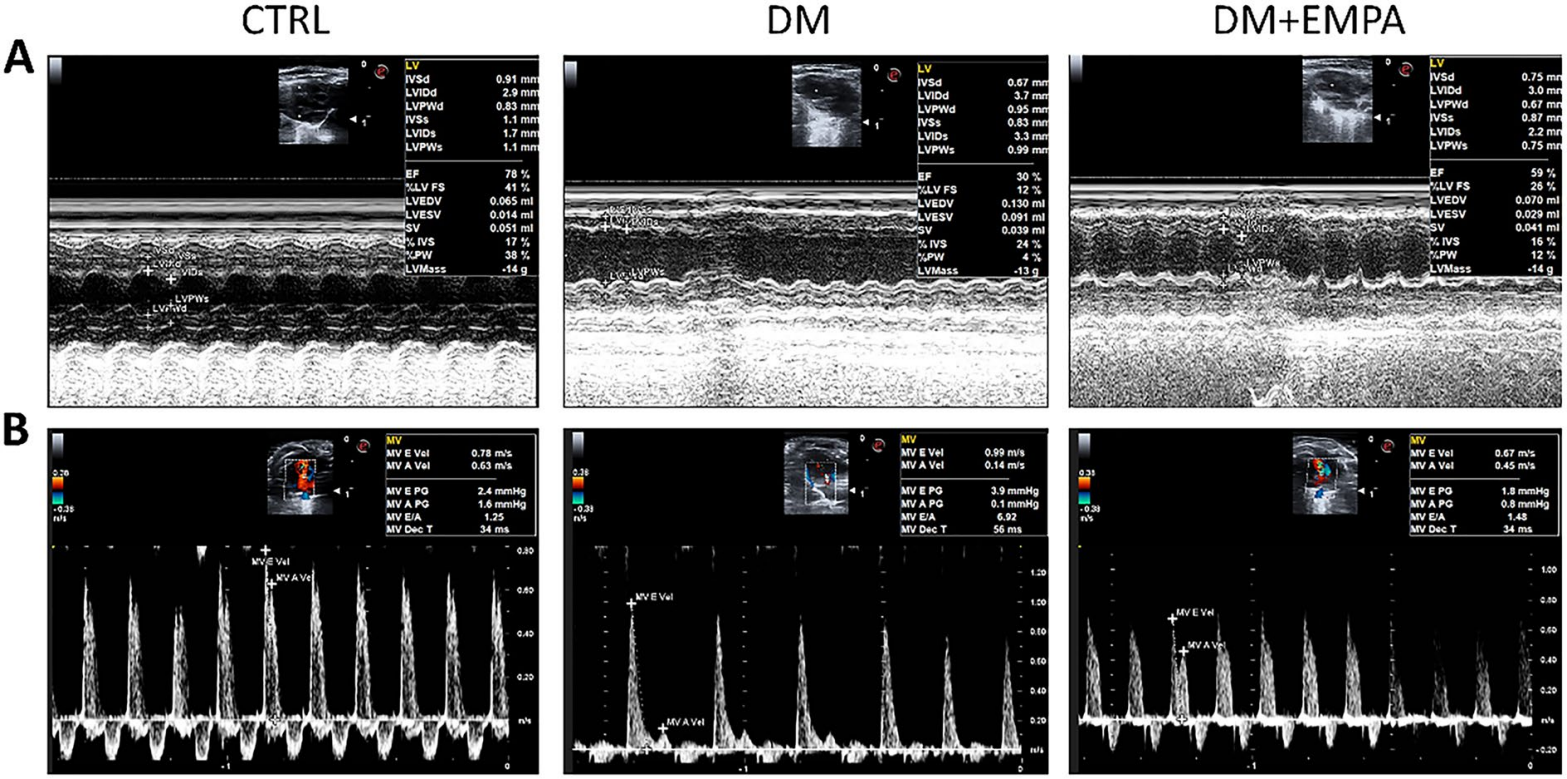
The structure and function of the hearts were examined by ultrasound. **A** showed the structural parameters of the hearts. **B** showed the diastolic function of the hearts

Patients with diabetes undergo structural changes in the heart, cardiac systolic and diastolic dysfunction, and heart failure [15]. We performed a multidimensional assessment of the structure and function of the mouse heart with ultrasonography, biomarkers, and pathology. Ultrasound and biomarker results showed that the left ventricle diameter and volume of the DM group increased, and the cardiac systolic and diastolic functions (E/A) decreased significantly compared with those of the CTRL group. As biomarkers of myocardial injury, serum hs-cTnT and NT-proBNP levels markedly increased. The effects of empagliflozin on diabetes-induced cardiac function were evaluated by using M-mode and Doppler echocardiography (Fig. 2). The systolic function of mice in the DM group was slightly lower (*P* < 0.05), and the left ventricular diameter and volume of mice in the DM group were significantly higher (*P* < 0.05), compared with those of the CTRL group (Table 1). Furthermore, the increase in mice's left ventricular diameter and volume were significantly inhibited by empagliflozin treatment (*P* < 0.05). However, there were no significant differences in the values of thickness of ventricular walls among the three groups. In addition, the expression of cardiac function biomarkers, NT-proBNP and hs-cTnT, increased in the DM group (*P* < 0.01), and empagliflozin treatment attenuated their expression (*P* < 0.01).

**Table1:** Cardiac structure and function data after 16 weeks of treatment in mice

	CTRL	DM	DM + EMPA	*P*
IVSd (mm)	0.96 ± 0.10	0.80 ± 0.13	0.86 ± 0.11	0.440
LVIDd (mm)	3.70 ± 0.57	4.06 ± 0.05	3.68 ± 0.43	0.339
LVPWd (mm)	0.97 ± 0.11	0.82 ± 0.17	0.88 ± 0.15	0.646
LVEDV (ml)	0.14 ± 0.05	0.17 ± 0.01	0.13 ± 0.04	0.313
LVESV (ml)	0.05 ± 0.03	0.1 ± 0.01*	0.06 ± 0.02^#^	0.027
EF (%)	65.00 ± 9.90	37.60 ± 6.25**	52.20 ± 6.76^#^	0.005
FS (%)	31.67 ± 7.04	15.40 ± 2.65**	22.60 ± 3.38^##^	0.005
E/A	1.30 ± 0.14	5.07 ± 2.83	2.26 ± 1.01	0.068
NT-proBNP (pg/mL)	309.94 ± 32.37	816.38 ± 32.51**	490.49 ± 36.38^##^	0.000
Hs-cTnT (pg/mL)	180.08 ± 7.53	297.41 ± 8.11**	228.68 ± 15.58^##^	0.000

IVSd: interventricular septal width during end-diastole, LVIDd: Left ventricular internal systolic diameter, LVPWd: Left ventricular posterior wall thickness during diastole, LVEDV: left ventricular end-diastolic volume, LVESV: left ventricular end-systolic volume, EF: left ventricle ejection fraction, FS: left ventricle fractional shortening, E/A: the ratio between early (E)-to-late (A) diastolic mitral inflow. Data were expressed as the mean ± SEM

*P < 0.05 versus CTRL; **P < 0.01 versus CTRL; ^#^*P* < 0.05 versus DM; ^##^*P* < 0.01 versus DM

### Empagliflozin improved diabetes-induced myocardial pathological changes

**Fig. 3:**
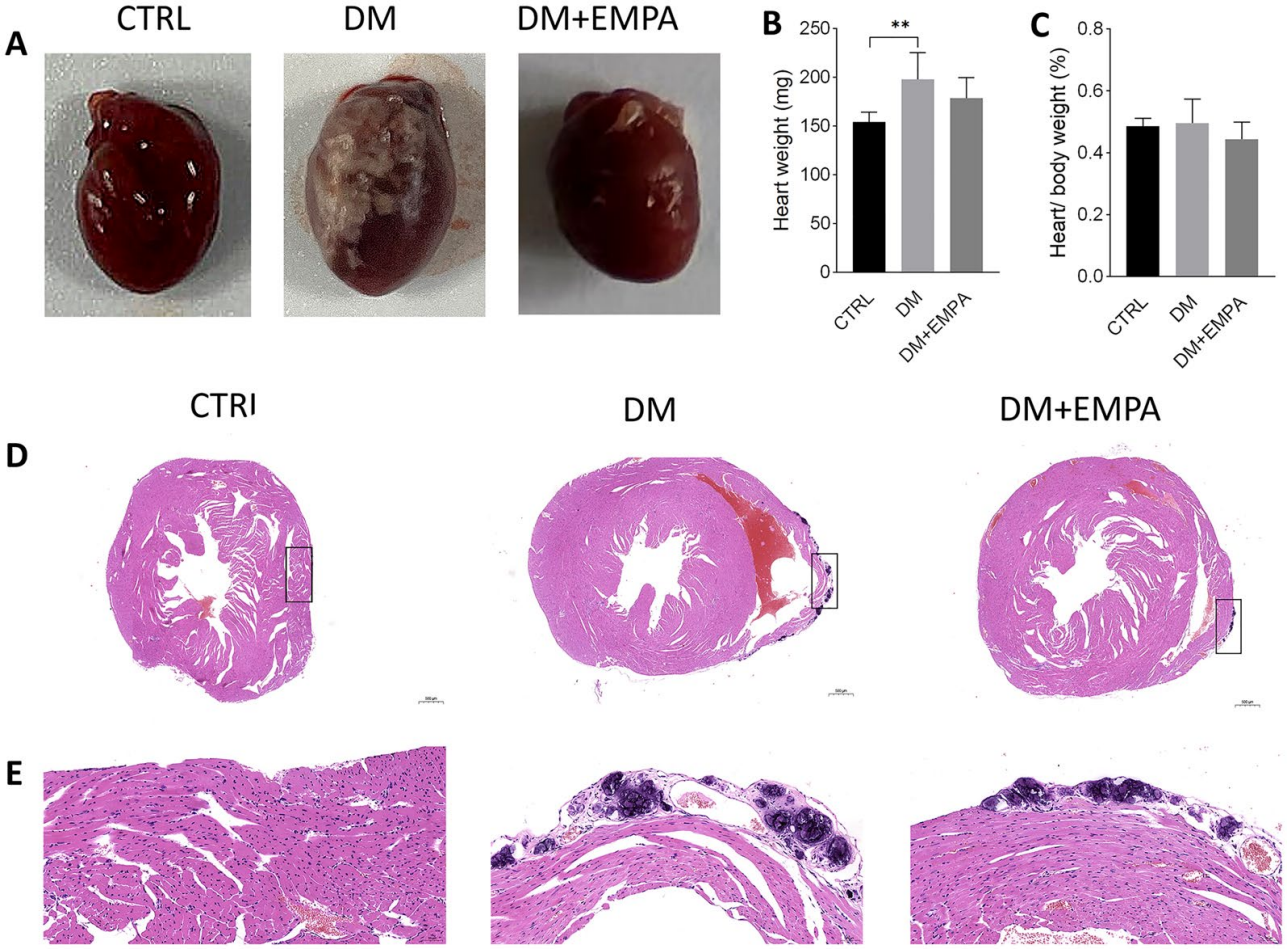
Effect of empagliflozin on myocardial pathological changes in diabetic mice. **A** showed freshly harvested mouse hearts under naked eyes. **B**, **C** showed the heart weight and the ratio between heart and body weight. **D**, **E** were H&E staining of the cross-section of mouse hearts. Data were expressed as the mean ± SEM. **P < 0.01 versus CTRL

**Fig. 4:**
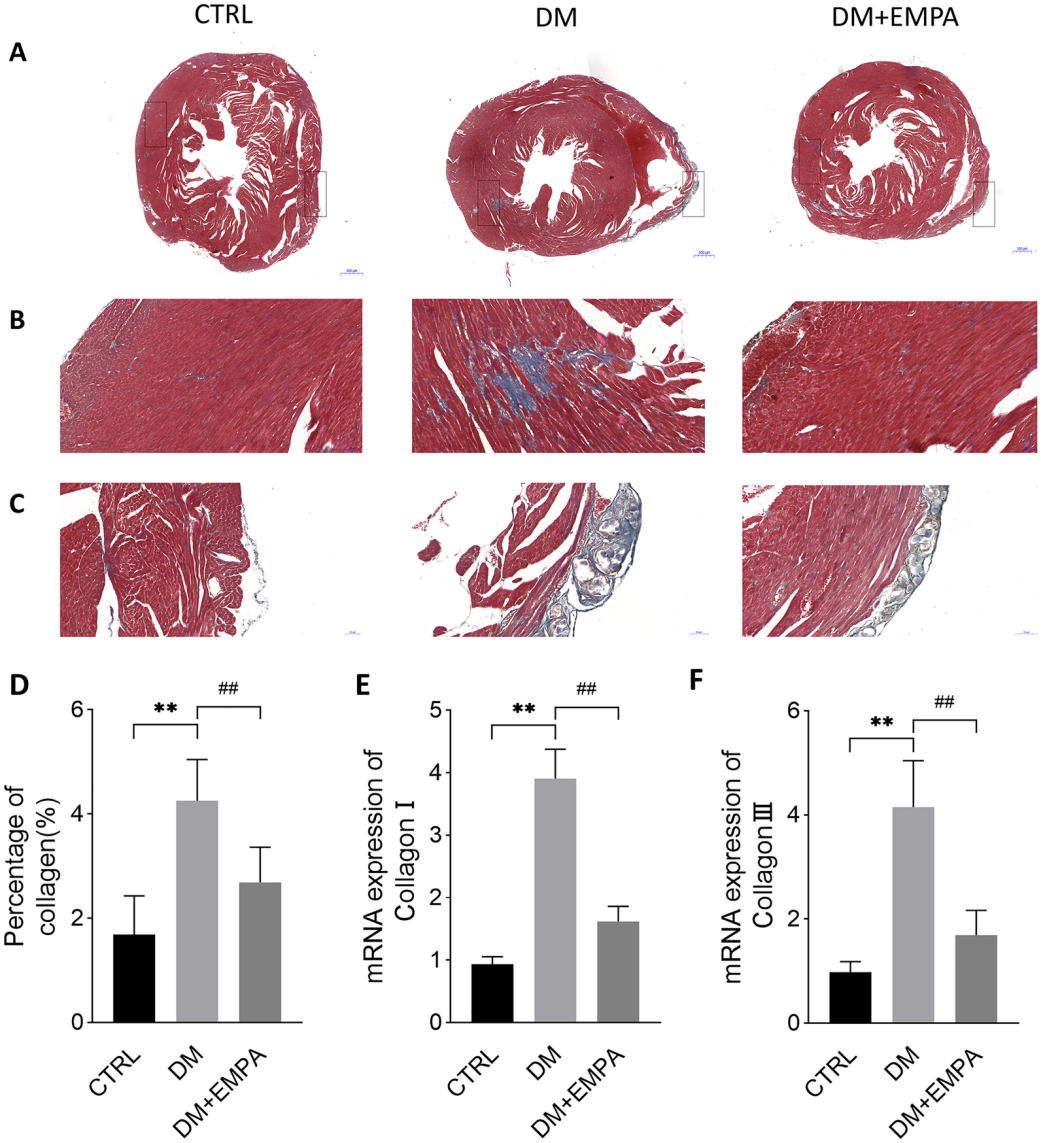
Effect of empagliflozin on myocardial fibrosis in diabetic mice. **A**–**C** showed Masson staining of the mouse heart cross-section, corresponding to the fibrosis of the global heart, the interstitial myocardium, and the epicardium, respectively. **D** showed the percentage of collagen in **A**. **E**, **F** showed mRNA of collagen I and collagen III in cardiac tissue. Data were expressed as the mean ± SEM. **P < 0.01 versus CTRL; ^##^*P* < 0.01 versus DM

The whole heart morphology showed that the DM group mice had a higher proportion of epicardial yellow-white fibrous connective tissueThe degree of change in the DM + EMPA group was between the other two groups (Fig. 3a). To investigate the effect of empagliflozin treatment on myocardial pathological changes, we performed H&E and Masson staining. H&E staining revealed that the cardiac tissue structure was clear in the CTRL group without obvious abnormality. In the DM group, the ventricle was dilated, with more foci of calcification, connective tissue hyperplasia, and punctate lymphocytic infiltration. In contrast, the mouse hearts in the DM + EMPA group had relatively normal cardiac structure, and function significantly attenuated myocardial cell arrangement disorder, epicardial connective tissue hyperplasia, and inflammatory cell infiltration (Fig. 3d, e). To assess the effect of empagliflozin on the amelioration of myocardial fibrosis, Masson staining and RT-PCR were performed to determine the degree of collagen deposition. The Masson staining indicated that the DM mice had excess collagen matrix accumulation in the myocardial tissues. Empagliflozin treatment significantly attenuated myocardial fibrosis in diabetic mice (Fig. 4).

### Effect of empagliflozin on myocardial proteomics in diabetes mice

**Fig. 5:**
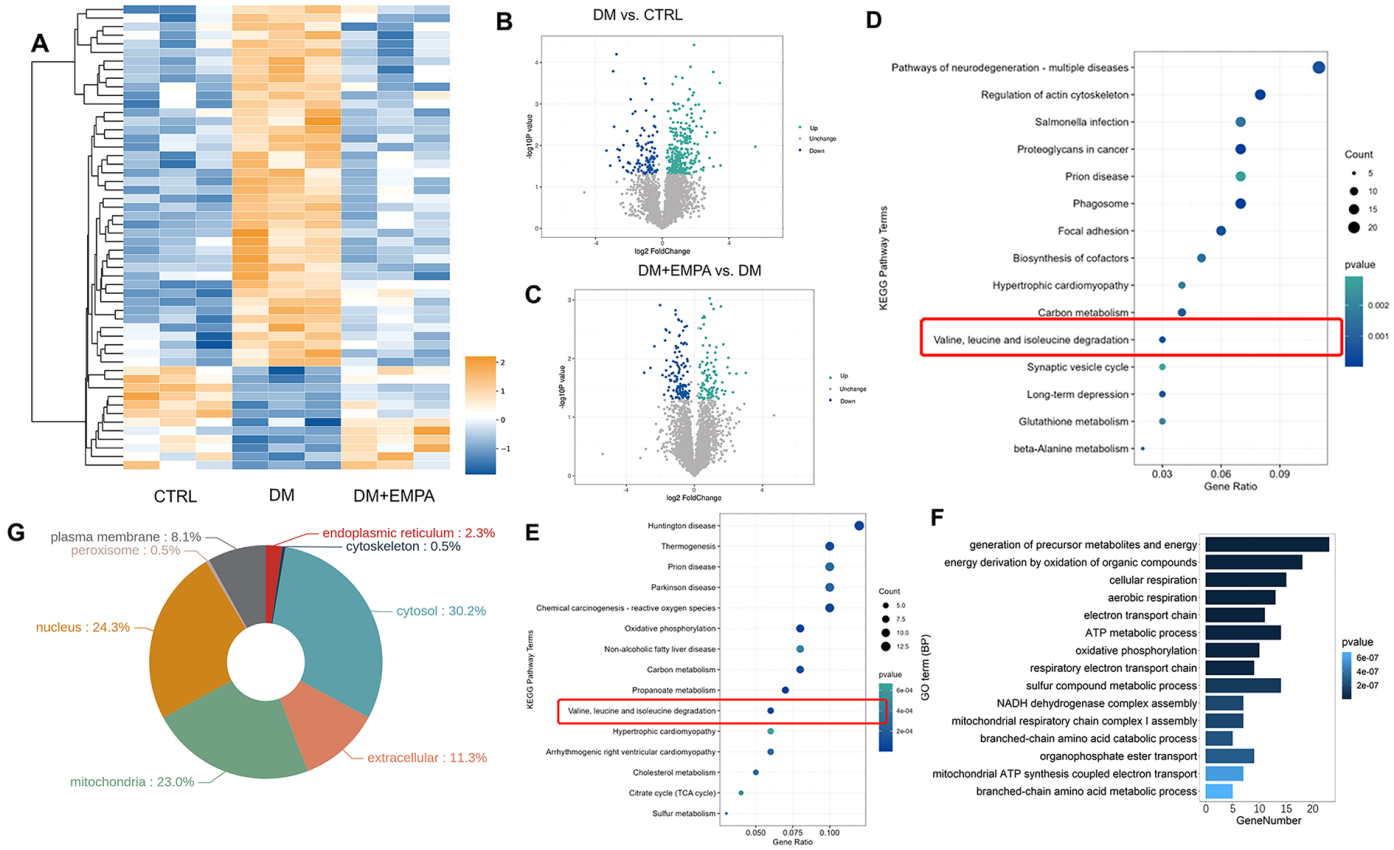
Bioinformatics analysis of differentially expressed proteins among three groups. **A** Heatmap showed the opposite expression of the proteins in the DM and DM + EMPA groups compared to the CTRL group. **B**, **C** Volcano maps illustrated the DEPs compared between the CTRL and DM groups and between the DM + EM and DM groups. **D**, **E** represented the KEGG pathway analysis of DEPs compared between the CTRL and DM groups and between the DM + EM and DM groups. **F** GO analysis of DEPs between the DM + EM and DM groups. BP: biological process, MF: molecular function. **G** Pie plots illustrated the subcellular localization of DEPs in the DM group and DM + EMPA group

By comparing the mass spectrometry results, 54 differentially expressed proteins (DEPs) were identified whose expression changed in opposite directions when the three groups were compared in pairs (Fig. 5a). DEPs analysis showed that diabetes could influence the expression of 417 proteins, with 308 up-regulated and 109 downregulated proteins compared with the CTRL group; empagliflozin impacted 223 proteins, with 101 up-regulated and 122 downregulated proteins compared with the DM (Fig. 5b, c). Most DEPs were primarily involved in metabolic processes such as energy and metabolism (Fig. 5e, f). GO and KEGG analyses showed that DEPs were relevant to the metabolic processes of various substances, including BCAA (Fig. 5d, e). In addition, the results of the subcellular localization analysis showed that the DEPs were localized in the cytosol (30.2%), nucleus (24.3%), and mitochondria (23.0%) (Fig. 5g).

### Empagliflozin promoted BCAA degradation in diabetic cardiomyopathy

**Fig. 6:**
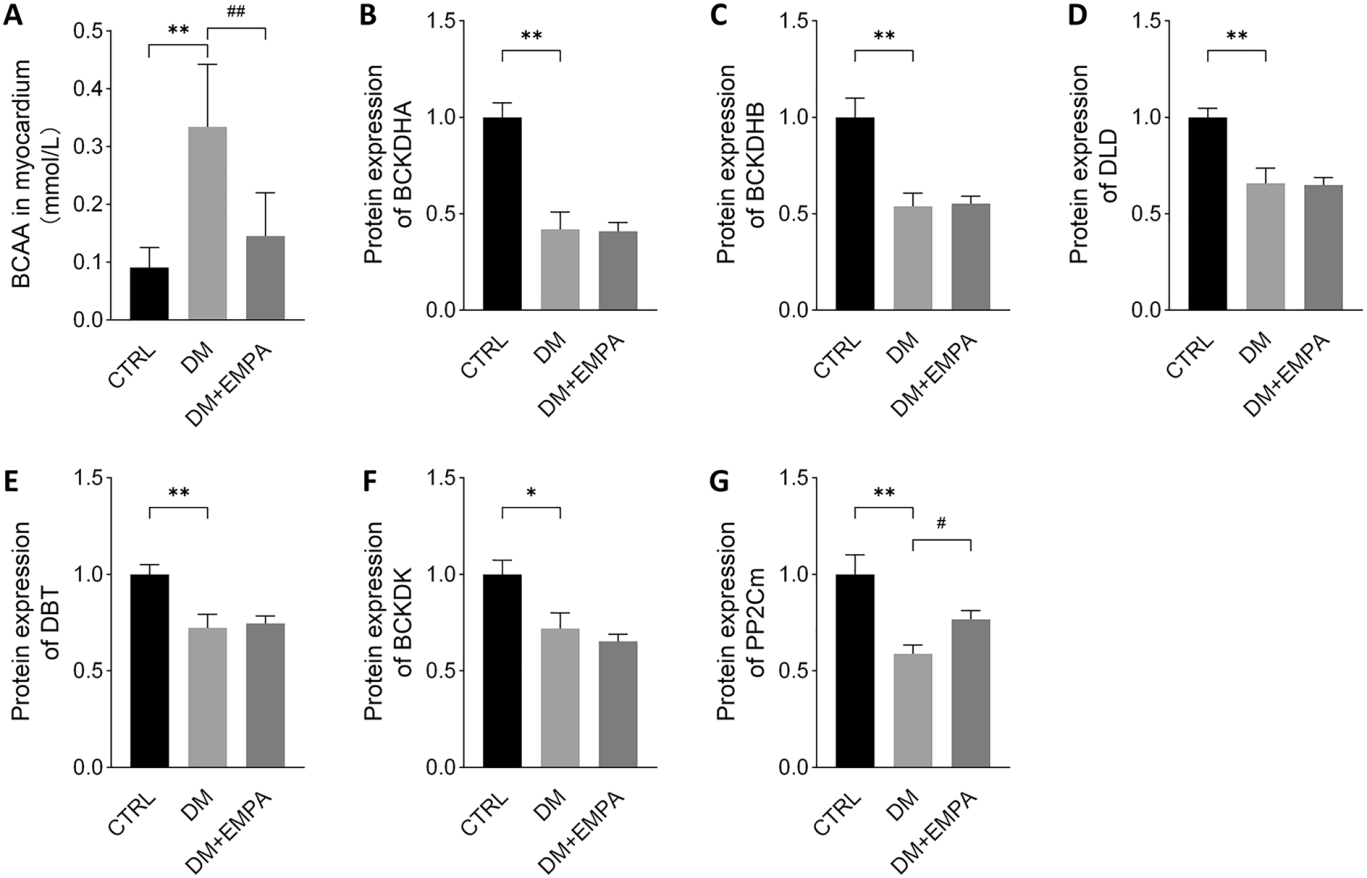
Effects of empagliflozin on BCAA degradation in diabetic cardiomyopathy. **A** Measurement of BCAA concentration in the myocardium. **B**–**G** Protein expressions of BCKDHA, BCKDHB, DLD, DBT, BCKDK, and PP2Cm in the mice myocardium were identified by PRM. *P < 0.05 versus CTRL; **P < 0.01 versus CTRL; ^#^*P* < 0.05 versus DM; ^##^*P* < 0.01 versus DM

We decided to take the BCAA degradation pathway as the object of follow-up research for three reasons, 1. differential expression of multiple proteins in the BCAA degradation pathway occurs; 2. BCAA is closely associated with diabetes and cardiovascular disease, and 3. the direct relationship between EMPA and cardiac BCAA metabolism has not been investigated. First, we determined that the concentrations of BCAA in both myocardial tissue and serum of the three groups of mice had indeed changed (Fig. 6a). Branched-chain ketoacid dehydrogenase (BCKDH), which is composed of BCKDHA, BCKDHB, DLD, and DBT, is the key enzyme involved in BCAA catabolism. It can be activated by PP2Cm and inhibited by BCKDK. To further determine the effect of empagliflozin on BCAA degradation in diabetic mice, we measured the levels of these six proteins in mouse hearts by PRM. As expected, the expression of the above enzymes in the DM group was significantly decreased compared with the CTRL group. Although, there was no statistical difference in the expression of BCKDHA, BCKDHB, and BCKDK between the DM and DM + EMPA groups (Fig. 6b–f). The expression of PP2Cm in myocardial tissue was increased in the DM + EMPA group, compared with that in the DM (Fig. 6g). These results indicated that empagliflozin promotes BCAA degradation through the up-regulation of PP2Cm.

### Effect of empagliflozin on mTOR/ULK1 signaling pathway in diabetic cardiomyopathy

**Fig. 7:**
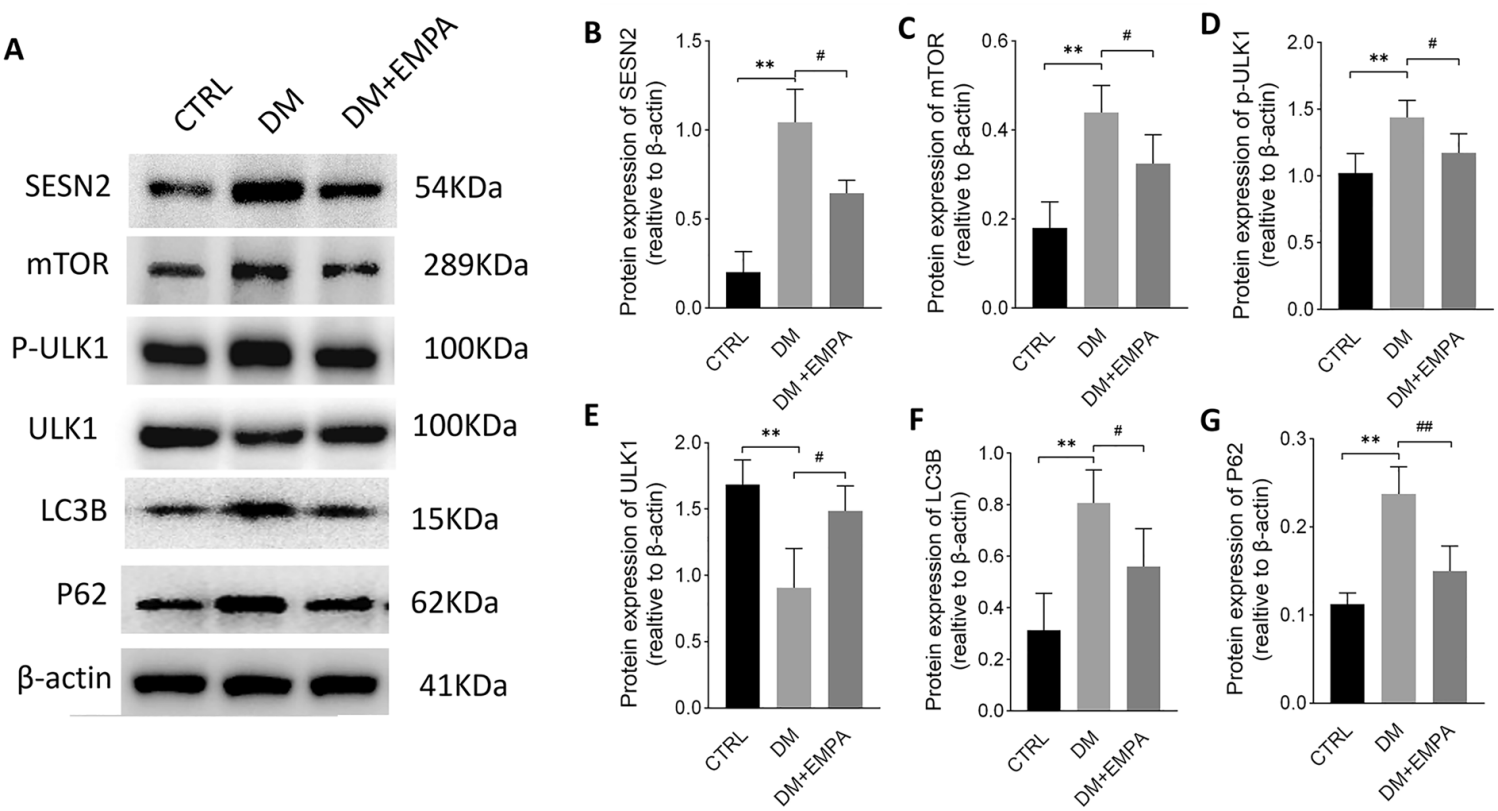
Empagliflozin promotes cardiac autophagy in diabetic mice through the mTOR/p-ULK1 pathway. **A** Protein expressions of SESN2, mTOR, p-ULK1, ULK1, LC3B, and P62 were identified by Western blotting. β-actin was considered as a loading control. **B**–**G** Data were expressed as the mean ± SEM. *P < 0.05 versus CTRL; **P < 0.01 versus CTRL; ^#^*P* < 0.05 versus DM; ^##^*P* < 0.01 versus DM

mTOR signaling pathway and autophagy play a significant role in the occurrence and development of DM. Previous studies have reported that leucine in BCAA is a potent activator of the mTOR pathway, which activates mTORC1 through direct binding to SESN2. Western blot results showed that elevated BCAA levels increased SESN2 and up-regulate mTOR in the diabetic myocardium (Fig. 7a–c). By phosphorylation of ULK1, mTOR inactivates the autophagy regulatory complex, thus affecting autophagosome biogenesis. ULK1 is a downstream enzyme in the mTOR signaling pathway that initiates autophagosome formation, and its activation is crucial for autophagy initiation [16]. In the DM group, the expression of ULK-1 decreased, and the expression of autophagy marker LC3B and autophagy substrate protein p62 increased, indicative of decreased autophagy activity. However, in the DM + EMPA group, the above changes were reversed. Due to decreased mTOR expression, ULK1 phosphorylation was reduced, autophagy was activated, autophagy-related protein ULK1 expression was increased, and LC3B and P62 were decreased.

## Discussion

Many previous studies and clinical trials have yielded substantial evidence of cardiovascular protection for SGLT2i. The benefits of SGLT2i in heart failure were reported to be consistent across the glycemic range [17, 18]. Moreover, other antidiabetic drugs, such as metformin, do not bring similar cardiovascular benefits under the same hypoglycemic effect. Therefore cardiac protection goes far beyond reduced serum glucose levels. The widely accepted empagliflozin may play a role in three ways: (1) improving the downstream oxidative stress and mitochondrial function by up-regulating Sirtuin, (2) blocking the sodium hydrogen exchange to reduce cardiac injury and pressure load, and (3) reducing epicardial lipid deposition, inflammation-mediated microenvironment dysfunction, and cardiac fibrosis [19]. Metabolic change is often regarded as an early marker of heart failure. There is still a broad scope for exploration of the effect of empagliflozin on myocardial metabolic changes.

Empagliflozin improves diabetes-related heart injury in multiple dimensions. First, our study was consistent with previous studies, demonstrating empagliflozin's significant hypoglycemic effect. Secondly, in this study, ultrasonic, pathological, and biomarker results showed that empagliflozin 3.75 mg/kg/d for 16 weeks improved the changes in cardiac structure and function and heart failure caused by diabetes. Our change is more pronounced than the treatment of 10 mg/d for 8 weeks in the same animal model [20]. The influence on the changing trend of NT-proBNP and hs-cTnT levels in the mouse experiment was consistent with the results of the EMPEROR study [21, 22]. Myocardial fibrosis is characterized by excessive deposition of extracellular matrix proteins, inducing myocardial stiffness and even left ventricular dysfunction, eventually leading to heart failure and death, which are common features of DM [23]. Consistent with the results of other studies, empagliflozin significantly improved myocardial fibrosis [24, 25]. In conclusion, empagliflozin improves diabetic systolic function and myocardial fibrosis.

We analyzed DEPs in the three groups using proteomic and biochemical analyses to further explore empagliflozin's not well-researched protective mechanism. We found that many DEPs were associated with material and energy metabolism. KEGG analysis revealed that six DEPs were enriched in the BCAA degradation pathway. Growing evidence suggests that BCAA degradation abnormalities and BCAA accumulation are important pathogenic mediators in the development of cardiovascular and metabolic diseases, including arteriosclerosis, coronary heart disease, hypertension, dilated cardiomyopathy, heart failure, and diabetes [26–30]. However, there have been no studies on SGLT2i and BCAA metabolism in the cardiovascular field. Our results show that empagliflozin-treated diabetic mice had up-regulated PP2Cm expression and decreased myocardial BCAA accumulation.

The mechanism by which BCAA affects cardiac function has not been thoroughly studied. The abnormality of cardiac BCAA catabolism is not likely to affect the energy supply in heart failure per se because BCAA oxidation constitutes a negligible amount (< 5%) of ATP production, even in a healthy heart [31]. Thus, it is more likely that altered concentrations of BCAA participate in the disease process by influencing signal transduction. BCAA is a potent activator of the mTOR pathway. The three BCAAs, leucine, isoleucine, and valine, are always eaten and combusted together, the relative abundance of which is nearly always approximately 2.2: 1.0: 1.6, reflecting the linked nature of their synthesis and oxidation. Therefore, detecting the total amount of BCAA can reflect the changing trend of a single BCAA. Leucine (but not valine nor isoleucine) promotes the mTOR signaling pathway by directly binding to SESN2, a negative regulator of mTORC1 activity [32]. It has been reported that partial genetic or pharmacological inhibition of mTORC1 reduces cardiac remodeling and heart failure [33–35]. In our study, SESN2/mTOR increased significantly in the DM group. In contrast, SESN2/mTOR becomes inactive in response to nutrient starvation, energy stress, hypoxia, or cellular damage [36]. Accordingly, in the DM + EMPA group, a significant regressive change was observed. Sun X et al. also found that empagliflozin could improve the cardiac function of obese mice by regulating the SESN2-mediated AMPK-mTOR pathway, which is solid evidence for our findings [32, 37].

Autophagy, a degradation mechanism of cytoplasmic components in cells, is an essential regulatory mechanism that maintains homeostasis and function of the heart under baseline conditions, which is activated during stress, limiting damage under most conditions [38]. Previous studies have shown that autophagy plays a protective role in attenuating cardiac fibrosis [39]. In many mouse models of type 2 diabetes, cardiac autophagy is impaired at the level of autophagosome formation or autophagosome-lysosome fusion [40]. Leucine in BCAA is mainly involved in autophagy, which is activated under amino acid deficiency and nutrient depletion. It was reported that increased cellular intake of leucine activated mTORC1-S6K1 and inhibited autophagy, while conversely, reduced leucine or the addition of rapamycin inhibited mTOR signaling and activated autophagy [41]. Liu et al. found that leucine deprivation in HEK 293 T cells' medium increased LC3B transformation and decreased p62, while supplementation of 10 mM leucine restored autophagy activity to the basic level, suggesting that leucine inhibited autophagy [42].

The mTOR pathway, which senses cellular nutritional and energy status, is a master regulator of several crucial cellular processes [36, 43]. ULK1, a critical molecule downstream of the mTOR signaling pathway, mediates the formation of autophagosomes, a key initial event in autophagy [16]. mTORC1 phosphorylates Ulk1 at serine 757, thereby inhibiting the formation of autophagosomes [44]. In this study, myocardial BCAA accumulation in DM group resulted in mTOR/p-ULK1 activation, increased LC3B (LC3B-I), and P62 expression, and impaired autophagy flow in the heart. However, empagliflozin treatment decreased the expression of these proteins and reactivated autophagy. In addition, Son et al. showed that leucine inhibited autophagy by acetylating the raptor component of mTORC1 [45]. The above experimental results confirm that mTOR is a crucial negative regulator of autophagy, and the reduction of BCAA levels could inhibit mTOR and enhance autophagy.

Empagliflozin may be an effective way to improve BCAA metabolism. BCAA is considered a biomarker of various cardiovascular diseases, therefore reducing BCAA levels may be a target for the treatment of cardiovascular diseases. The therapeutic impact of targeting BCAA catabolic flux under pathological conditions has not been adequately investigated. Reducing BCAA content in the body may be carried out in two ways: reducing intake and increasing excretion. However, a UK twin cohort study found no association between BCAA intake and plasma concentration. Plasma BCAA levels did not directly reflect dietary source intake, as patients with higher BCAA intake had a lower prevalence of excessive weight, insulin resistance, systemic inflammation, and hypertension [46]. Therefore, the accumulation of BCAA may be caused mainly by abnormal decomposition rather than excessive intake. Identifying therapies that target the degradation of BCAAs may be of great significance. The treatment options for improving BCAA degradation are limited. Previous studies have shown that 3,6-dichlorobenzo[b] thiophene-2-carboxylic acid (BT2) promotes BCAA degradation by inhibiting BCKDK, increasing the activity of BCKDH and effectively alleviating stress-induced heart failure [47, 48]. Before BT2 can enter the clinic as a drug, more studies must be conducted. In addition, pyridostigmine, an anticholinesterase drug, was found to promote BCAA catabolism by affecting the vagal activity and gut microbiota [49], which definite anticholinesterase toxicity hinders its wide application in the cardiovascular field. However, the results of our study showed that empagliflozin, a mature oral medication, could also contribute to alteration. Empagliflozin treatment significantly increased PP2Cm expression, improved the efficiency of BCKDH and reduced BCAA accumulation in tissues. It has been reported that the overexpression of PP2Cm could promote BCAA metabolism and reduce heart failure in myocardial infarction models, which also strongly supports our findings [50]. Gong et al. showed that in diabetic retinopathy models, empagliflozin inhibited BCKDK expression and promoted BCAA metabolism [11]. The organ-specific expression of PP2Cm may cause different regulatory modes of empagliflozin on BCAA degradation. According to Genecard, PP2Cm expresses in the myocardium but not the retina.

**Fig. 8:**
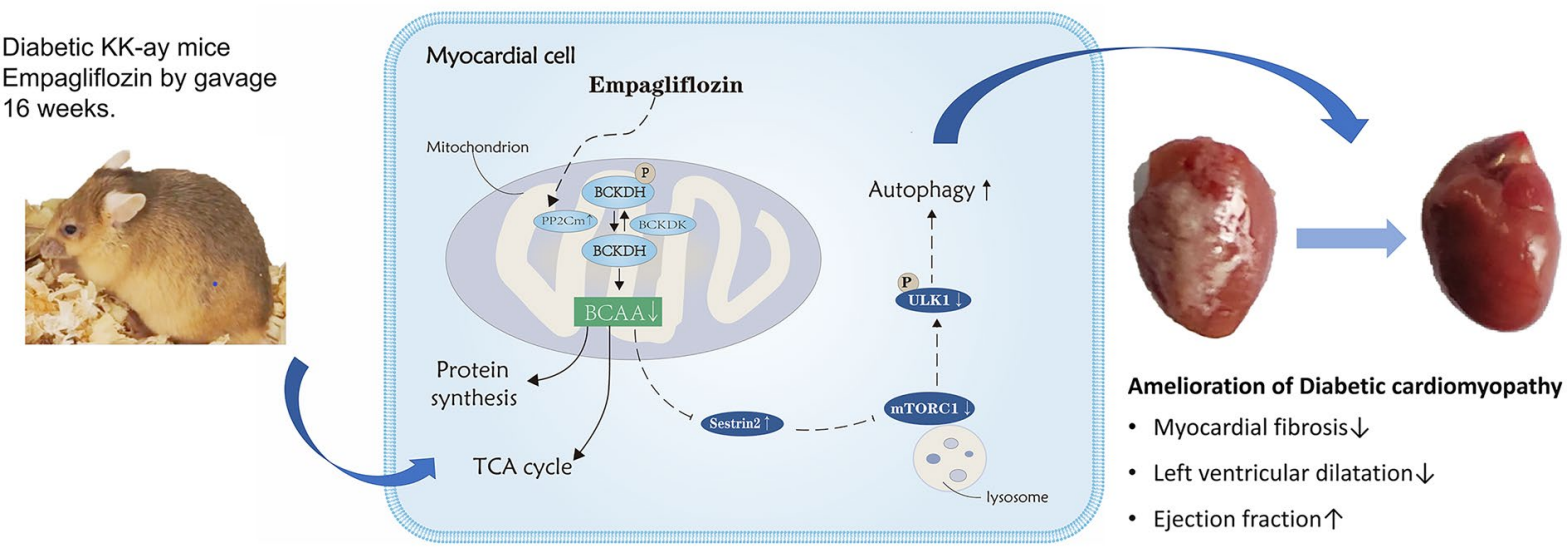
Graphical abstract. Empagliflozin attenuated diabetic cardiomyopathy-related myocardial fibrosis and heart failure through up-regulating PP2Cm, promoting catabolism of BCAA, down-regulating mTORC1, inhibiting p-ULK1, and increasing autophagy. Solid arrows indicate activation, T-arrows indicate inhibition, and dotted arrows indicate indirect effect

In conclusion, our study indicated that empagliflozin could protect diabetic cardiomyopathy by up-regulating PP2Cm, promoting catabolism of BCAA, and downregulating mTOR/p-ULK1, thereby up-regulating ULK1 and increasing autophagy. These findings suggest that empagliflozin could be a potential candidate drug against BCAA increase (Fig. 8).

Because of the similar metabolic characteristics of BCAA in various cardiovascular diseases, whether empagliflozin and other SGLT2 inhibitors could promote BCAA degradation in other cardiovascular diseases through similar mechanisms and how empagliflozin up-regulates PP2Cm remains to be revealed in further studies. Empagliflozin may have the potential to be used in cardiovascular diseases characterized by abnormal BCAA metabolism.

## Data Availability

Data will be made available on request.

## Abbreviations

DM: Diabetic cardiomyopathy
BCAA: Branched-chain amino acid
SGLT2i: Sodium–glucose co-transporter 2 inhibitor
mTOR: Mammalian target of rapamycin
TG: Triglycerides
TC: Total cholesterol
HDL: High-density lipoprotein cholesterol
LDL: Low-density lipoprotein cholesterol
DEPs: Differentially expressed protein
BCKDH: Branched-chain ketoacid dehydrogenase
BCKDK: Branched-chain keto acid dehydrogenase kinase
